# Ethical Considerations in Research With People From Refugee and Asylum Seeker Backgrounds: A Systematic Review of National and International Ethics Guidelines

**DOI:** 10.1007/s11673-023-10297-w

**Published:** 2023-10-27

**Authors:** Natasha Davidson, Karin Hammarberg, Jane Fisher

**Affiliations:** Global and Women’s Health, School of Public Health and Preventive Medicine, Monash University Faculty of Medicine Nursing and Health Sciences, 553 St Kilda Road, Melbourne, Victoria 3004 Australia

**Keywords:** Research Ethics, Systematic Review, Vulnerable populations, Refugees, Guidelines

## Abstract

Refugees and asylum seekers may experience challenges related to pre-arrival experiences, structural disadvantage after migration and during resettlement requiring the need for special protection when participating in research. The aim was to review if and how people with refugee and asylum seeker backgrounds have had their need for special protection addressed in national and international research ethics guidelines. A systematic search of grey literature was undertaken. The search yielded 2187 documents of which fourteen met the inclusion criteria. Few guidelines addressed specific ethical considerations for vulnerable groups much less people with refugee and asylum seeker backgrounds. One guideline explicitly addressed vulnerability for refugees and asylums seekers. To ensure members of ethics committees and researchers consider the potential challenges of conducting research with these groups, guidelines may need to be supplemented with a refugee and asylum seeker specific research ethics framework. Such a framework may be necessary to optimally protect people with refugee and asylum seeker backgrounds in research.

## Introduction

Research ethics standards guide researchers and aim to protect research participants from harm. The ethics principles that underpin the conduct of biomedical and behavioural research involving human participants stem from the Declaration of Helsinki (World Medical Association 2001). This Declaration provides a set of ethics principles regarding human research to safeguard the health of participants. It was developed by the World Medical Association and adopted in 1964, and is accepted internationally as a cornerstone of research ethics (Bosnjak 2001, World Medical Association 2013). The Declaration of Helsinki was followed in 1979 by the United States Department of Health, Education, and Welfare’s Belmont Report (The Belmont Report) (The National Commission for the Protection of Human Subjects of Biomedical and Behavioral Research 1979) which identifies three basic ethical principles that underlie research involving humans: respect for persons, beneficence, and justice. These three principles respectively guide researchers to respect for the autonomy of potential and actual research participants; ensure that the benefits of their research outweigh the risks; and that the benefits, risks and burdens of research are fairly distributed (The National Commission for the Protection of Human Subjects of Biomedical and Behavioral Research 1979). Research ethics guidelines recommend researchers use standard practices to put each principle into practice (National Health and Medical Research Council 2007).

Respect for persons encompasses two moral requirements: to acknowledge an individual’s autonomy and to protect those with diminished autonomy (The National Commission for the Protection of Human Subjects of Biomedical and Behavioral Research 1979). Obtaining voluntary informed consent, either verbally or in writing, is the standard procedure for operationalizing the principle of respect. It always involves providing prospective research participants with the opportunity to consent or decline their invitation to participate voluntarily, that is in the absence of coercion (use of force or a threat of harm to obtain compliance) or undue influence (non-threatening but excessive persuasion) (The National Commission for the Protection of Human Subjects of Biomedical and Behavioral Research 1979). To ensure a prospective participant decides voluntarily, researchers may provide prospective participants the time and opportunity to consult others before making a decision (Gupta 2013). They should strive to minimize the power inequalities between the person inviting and the prospective participant, because being relatively powerless diminishes autonomy. Relations of unequal power researchers should be aware of in voluntary informed consent procedures include those between teachers and students, doctors and patients, and warders and jailers (Nijhawan et al. 2013). For consent to be voluntary and informed, the person providing it must have the capacity to decide for themselves, that is the cognitive ability to make considered choices and to act in accordance with their own beliefs and values (Mackenzie, McDowell, and Pittaway 2007). Children (legal minors, in most countries young people under eighteen years of age) and people with cognitive impairments are considered unable to provide voluntary informed consent because of their limited cognitive capacity. Like being relatively powerless, having limited cognitive capacity diminishes autonomy and requires researchers to include special protections in their research design.

The principle of beneficence relates to balancing the risks and benefits of research, which may be physical, psychological, social, and/or material. An important component of beneficence is non- maleficence which refers to protecting individual participants as well as their communities, from unnecessary and foreseeable harms related to the research (The National Commission for the Protection of Human Subjects of Biomedical and Behavioral Research 1979). Achieving beneficence also requires research results in individual-or social-level benefits. In most research projects the key foreseeable benefit is scientific knowledge that can be used for social good (Aarons 2017; Resnik 2016). Ensuring research is scientifically robust is an important aspect of optimizing the benefits of research because poorly designed research cannot create reliable and beneficial evidence.

Individual level physical, psychological, social, or material/economic harms and benefits for participants vary by degree and type, depending on the nature of the research and the participants social situation. Physical harms such as adverse side effects from experimental drugs or procedures are the focus of biomedical research. Psychological and social harms receive more attention in social and behavioural research. Negative affective states associated with research such as anxiety, depression, guilt, shock, and loss of self-esteem are important psychological harms (National Health and Medical Research Council 2007). They typically arise as a result of reliving traumatic events in research interviews. Social harms include adverse changes in relationships, stigmatization, embarrassment, and reputational damage (National Health and Medical Research Council 2007). These harms are most likely to occur due to confidentiality breaches (e.g. a participant being identified in relation to a sensitive condition or behaviour they disclosed to the researcher) or negative representations of participants in research reports. Economic harms include direct and indirect costs associated with participation such as money spent on child care, as well as loss of economically productive time (Pieper and Thomson 2016). Risks of harm occurring in research need to be assessed and outweighed by the anticipated benefits. Benefits for individual participants may include improved physical or psychological health, positive new relationships (social) and financial rewards (e.g. payments to health volunteers—economic) (Fisher et al. 2018).

Undertaking a risk benefit analysis, to understand the magnitude of possible harms and anticipated benefits, is the standard procedure for operationalizing the beneficence principle. In addition to assessing the overall magnitude of risk and benefits, researchers ought to assess their distribution to ensure their research achieves the principle of justice.

The principle of justice requires procedures are fair in the selection of research participants and fair distribution of the risk and benefits of research (National Health and Medical Research Council 2007). This principle means ensuring that the people who take on the burden of participation (and/or people like them) have access to the benefits of research, including new programmes or therapies developed based on the results of the research. People from refugee or asylum seeker backgrounds may only ethically be targeted for participation if the research questions are focused on those groups and the results intended and likely to benefit them (Seagle et al. 2020; Block et al. 2013). Most research conducted with these populations are likely to benefit refugees and asylum seekers, since that research is about issues specific to those groups and the outcomes applicable to only those groups. In this review, we acknowledge the importance of person-first language but in the interests of brevity, throughout this paper we refer to people from refugee and asylum seeker backgrounds as “refugees and asylum seekers.” This term signifies the context of people from refugee and asylum seeker backgrounds and their experiences.

## Refugee and Asylum Seeker Populations’ Vulnerabilities and Structural Coercion

Vulnerability in research ethics is defined, as “a condition, either intrinsic or situational, of some individuals that puts them at greater risk of being used in ethically inappropriate ways in research” (National Bioethics Advisory Commission 2001, 85). Ethically inappropriate treatment is most likely to occur when an individual’s autonomy to decide about research participation for themselves is undermined. In addition to the overt threats of harm traditionally associated with coercion, some populations such as refugees or asylum seekers are more likely to experience what Fisher (2013) terms “structural coercion” (Fisher 2013).

This concept highlights how structural disadvantage related to the broader social, economic, and political context can compel individuals to participate in research (Fisher 2013). For example, socio-economically disadvantaged people, including refugees and asylum seekers, with limited access to medical treatment may feel compelled to enrol in research to access a treatment they cannot otherwise afford (Ravinetto et al. 2015). Coercion is related to the threat of harms that may occur in the absence of treatment. Structural economic disadvantage undermines autonomy in people with adequate cognitive capacity to decide for themselves. This contrasts with other groups such as children or people with intellectual disabilities, whose lack of autonomy to consent stems from their limited cognitive abilities (Gordon 2020).

Refugees and asylum seekers need for special protections in research are underpinned primarily by the relative powerlessness associated with the challenges experienced pre and post arrival in a resettlement country. They typically have a history of pre-migration experiences in their country of origin and in transit such as displacement, political persecution, systematic and severe discrimination and economic hardship. Those who have been forced to migrate as refugees or asylum seekers have often suffered serious physical, psychological, and emotional trauma and may have tenuous rights in their host country, for example those seeking asylum may have temporary rather than permanent residence (Silove, Ventevogel, and Rees 2017).

Refugees and asylum seekers may also experience challenges related to structural disadvantage after migration and during resettlement. These include racial or xenophobic discrimination, not being fluent in the language spoken in the host country and or economic hardship related to difficulty finding work (Ziersch et al. 2020). These disadvantages collectively leave refugees and asylum seekers in positions of relatively limited power and diminish their autonomy. While these challenges underscore the importance of conducting research that can inform the development of services and programmes targeting refugees and asylum seekers, they also create challenges for researchers as they attempt to design research that is ethical. These vulnerabilities require that researchers build special protections into research with refugees and asylum seekers to achieve the research ethics principle of autonomy.

## Refugees and Asylum Seekers Heightened Risk of Harm

Vulnerability in research may also be conceptualized in relation to the principle of beneficence. This stems from a person being at greater risk of harm than others in the same situation (Gordon 2020). Refugees and asylum seekers may have increased susceptibility to being harmed or wronged in research because of their structural disadvantage and/or their challenging historical circumstances (Fisher 2013). Structural factors, such as exclusion and lack of political representation, may mean refugees and asylum seekers are more likely to agree to put themselves at risk of harm by participating in research that involves greater risk (Seagle et al. 2020). They may also be more susceptible to harm. For example, refugees and asylum seekers may be more likely to experience psychological trauma from discussing past experiences in research, by virtue of the extremely traumatic experiences they have had (Silove et al. 2017).

Participation of refugees and asylum seekers in research, aims to ensure their representation in health policy and health services (Seagle et al. 2020). While refugees and asylum seekers are not necessarily more at risk of harm than other vulnerable groups (e.g. children, prisoners), their vulnerabilities stem from particular historic experiences and structural disadvantages. Because vulnerability has different underpinnings depending on individuals’ circumstances, measures taken to protect individuals deemed at risk of harm from participating in research need to be tailored. Against this backdrop, this research builds on Bracken Roache and colleagues’ (2017) review of the definitions, applications, and implications of vulnerability in research guidelines (Bracken-Roche et al. 2017). The aim of this review was to assess the extent to which refugees and asylum seekers have their special need for protection in research participation addressed in major national and international research ethics policies and guidelines. The aim was also to assess the level of consideration and provision for addressing the special needs for protection of refugees and asylum seekers in research participation and to identify any gaps or areas of improvement in existing research ethics policies and guidelines regarding their protection in research.

## Methods

To address the aim, a scoping review using a systematic search of the grey literature was conducted. It followed the Preferred Reporting Items for Systematic Reviews and Meta-Analysis recommendations for systematic searches (Rethlefsen et al. 2021).

### Inclusion Criteria

Guidance documents were included if they were a) English language research ethics guidelines, standards, policies, and regulations (henceforth referred to as guidelines). Further inclusion criteria were that the guideline was b) international or national in scope; and c) from a country that receives a predetermined annual allocation of refugees through the United Nations Resettlement Programs (Australia, Canada, the European Union (treated as one region), the United Kingdom and the United States) (UNHCR 2021) and international guidelines. No date limits were applied.

### Search Strategy

We adopted the systematic strategy for searching and retrieving “grey literature” in public health contexts described by Godin and colleagues (Godin et al. 2015). This strategy was based on a search plan which defined the resources to be searched, search terms, websites, and limits to be used, prior to conducting the search (Cumpston et al. 2019). The search plan which incorporated four methods for identifying relevant documents was developed: (1) keyword searching in Google search engine (2) reviewing content of targeted websites (3) hand-searching of the International Compilation of Human Research Standards (Office for Human Research Protections 2020) and (4) hand-search of the reference lists in documents identified in (1), (2), or (3). The search was conducted between 30th July and 30th August 2021.

The Google keyword search was conducted using the search string “ethics” AND “research” AND “guidelines” OR “policies” OR “standards”. The first 100 results were selected and screened. This number was chosen to capture the most relevant items while still being a feasible amount to screen (Godin et al. 2015). Targeted websites were identified and selected by reviewing screened documents. They included international health organizations (World Health Organization, United Nations agencies and the European Union) and national governments health websites (Australia, Canada, United States, United Kingdom). Lastly, national and international research and government organization websites identified in the International Compilation of Human Research Standards and reference lists in previously identified documents were hand searched (Office for Human Research Protections 2020).

### Screening

Since abstracts are often not included in grey literature documents, titles, executive summaries and table of contents were screened for relevance by the first author, ND. After removing documents that did not meet inclusion criteria, full texts of the remaining documents were reviewed. Key organizations provide international and national oversight of ethical standards with research participants. Statutory and legislative requirements are often encompassed in these standards. Hence, this review pertains to both binding and non-binding requirements or guidelines where ethics review boards and researchers seek guidance.

### Data Collection Process

Each guideline was reviewed for content relating to research ethics considerations of vulnerable populations in general and refugees and asylum seekers specifically. Given the concepts of interest, each guideline was word searched for “vulnerab” and “ethnic groups,” “racial minority” (which may include refugees and asylum seekers) and “refugees” and “asylum seekers.” Search terms were kept broad to increase the likelihood of identifying relevant content in the documents.

### Data Extraction

Guideline characteristics were extracted and manually entered into Microsoft Excel by the first author, ND using a proforma. The guidelines official name, the organization that had developed it, the year published, and the region or jurisdiction it applied to were recorded. Bracken Roache and colleagues (2017) data extraction strategy for assessing vulnerability content in research ethics guidelines (Bracken-Roche et al. 2017) was modified to focus on ethical considerations for research involving refugees and asylum seekers. The following data were extracted:

- whether vulnerability was described
- whether groups or individuals that are considered vulnerable were described
- whether the reasons why these population groups or individuals are considered vulnerable were described
- whether ethical considerations were linked to vulnerability (generally and more specifically)
- whether ethical considerations were linked to the needs of refugees and asylum seekers (generally and more specifically).

## Results

**Fig. 1:**
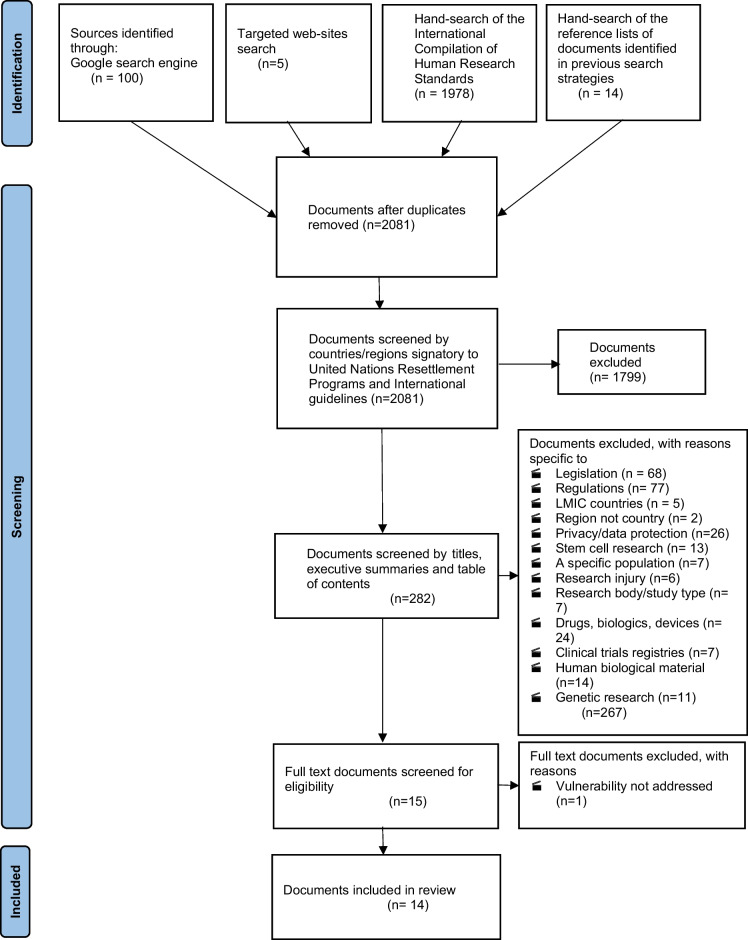
Flow diagram showing the process of study selection

Fourteen documents met the inclusion criteria. The Preferred Reporting Items for Systematic Reviews and Meta-Analyses flow diagram illustrating the systematic search for documents is presented in Figure 1. The guidelines titles and an overview of the ethical considerations relating to the need that they addressed, are described in Table 1.

**Table 1:** Content of ethical guidelines relating to research with vulnerable groups or individuals

**Guideline** **(Short title)** ***(Organization)***	**Year**	**Region**	**Abbreviation**	**1) Defined vulnerability, vulnerable groups or individuals**	**2) Described groups or individuals that are considered vulnerable**	**3) Described why these groups or individuals are considered vulnerable**	**4) Recognising ethical considerations are linked to vulnerability (generally and more specifically) (including how protections are implemented)**	**5) Recognising ethical considerations are linked to the needs of refugees and asylum seekers (generally and more specifically including how protections are implemented)**
***International***								
Guidance note — Research on refugees, asylum seekers & migrants (EU Guidance Note) *(EUROPEAN COMMISSION Directorate-General for Research and Innovation)*	2020	EU	EU Guidance Note	No	*"Research on refugees, asylum seekers and migrants concerns a particularly vulnerable group." (p.1)*	No	Yes	*GENERALLY "Research on refugees, asylum seekers and migrants concerns a particularly group which needs particular safeguards in terms of research ethics." (p.1)* *SPECIFICALLY Consider including researchers with a refugee or migrant background to mitigate potential risks of coercion or power differentials." (p.2)*
REGULATION (EU) No 536/2014 OF THE EUROPEAN PARLIAMENT AND OF THE COUNCIL of 16 April 2014 on clinical trials on medicinal products for human use (EU Clinical Trials Regulation) *(European Commission)*	2014	EU	EU Clinical Trials Regulation	No	*"...minors and pregnant or breast-feeding women" (p.20)*	No	*GENERALLY* minors and pregnant or breast-feeding women but has not described how the protections should be implemented*"...a clinical trial provides for the participation of specific groups or subgroups of subjects, where appropriate, specific consideration shall be given to the assessment of the subjects concerned." (p.20)*	No
Declaration of Helsinki (Helsinki Declaration) *(World Medical Association)*	2013	Int	Helsinki Declaration	No	*"Some groups and individuals are particularly vulnerable and may have an increased likelihood of being wronged or of incurring additional harm." (p.2)*	No	*GENERALLY "All vulnerable groups and individuals should receive specially considered protection." (p.2)*	No
Standards and Operational Guidance for Ethics Review of Health-Related Research with Human Participants (WHO Standards) *(World Health Organization (WHO))*	2011	Int	WHO Guidance for Ethics Reviews	*"Vulnerable (research) participants: Vulnerable persons are those who are relatively (or absolutely) incapable of protecting their own interests. More formally, they may have insufficient power, intelligence, education, resources, strength, or other needed attributes to protect their own interests." (p.41)*	*"…members of a group with a hierarchical structure, such as medical, pharmacy, dental and nursing students, subordinate hospital and laboratory personnel, employees of the pharmaceutical industry, members of the armed forces, and persons kept in detention. Others include patients with incurable diseases, people in nursing homes, unemployed or impoverished people, patients in emergency situations, ethnic minority groups, homeless people, nomads, refugees, minors, and those incapable of giving consent." (p.41)*	*"Individuals whose willingness to volunteer in a research study may be unduly influenced by the expectation, whether justified or not, of benefits associated with participation, or of a retaliatory response from senior members of a hierarchy in case of refusal to participate may also be considered vulnerable." (p.41)*	*GENERALLY "The primary task of an REC is the ethical review of research protocols to review the ethical acceptability of measures to ensure protection of vulnerable populations" (p.12). Other vulnerable persons include.....and those incapable of giving consent." (p.41)*	*GENERALLY "The primary task of an REC is the ethical review of research protocols to review the ethical acceptability of measures to ensure protection of vulnerable populations" (p. 12) "vulnerable persons include ….. ethnic minority groups, refugees." (p.41)*
UNESCO Universal Declaration on Bioethics and Human Rights (UNESCO Declaration) *(UNESCO)*	2005	Int	UNESCO Declaration	No	No	No	*GENERALLY "Individuals and groups of special vulnerability should be protected and the personal integrity of such individuals respected." (p.77)* *SPECIFCALLY* Capacity to consent *-"special protection is to be given to persons who do not have the capacity to consent" "Respect for human vulnerability and personal integrity. In applying and advancing scientific knowledge, medical practice and associated technologies, human vulnerability should be taken into account."(p.77)*	No
International Ethical Guidelines for Health-related Research Involving Humans (CIOMS) *(Council for International Organizations of Medical Sciences)*	2002	Int	Council for International Organizations	*“Vulnerability involves judgments about both the probability and degree of physical, psychological, or social harm, and a greater susceptibility to deception or having confidentiality breached....and they are relatively (or absolutely) incapable of protecting their own interests." (p. 57).*	*“Characteristics within groups can make it reasonable to assume that certain individuals are vulnerable.” (p.57)* Extensive examples of the vulnerable characteristics within population groups are given for each individual ethical consideration.	[Being vulnerable]* "may happen when people are marginalized, stigmatized, or face social exclusion or prejudice that increases the likelihood that others place their interests at risk, whether intentionally or unintentionally." (p.57)*	*GENERALLY Respect for Persons entails “protection of persons with impaired or diminished autonomy, which requires that those who are dependent or vulnerable be afforded security against harm or abuse”* *SPECIFICALLY* Capacity to consent -* "One criterion of vulnerability is limited capacity to consent or decline to consent to research participation." (p.57) Individuals in hierarchical relationships "possibility of diminished voluntariness of the consent of potential participants who are in a subordinate relationship" (p.58) Institutionalised persons where groups resident in “an inherently coercive environment and are denied certain freedoms.” (p.58) Women "specific circumstances for women could make women vulnerable." (p.58)*	*GENERALLY "To the extent that these and other people have one or more of the characteristics discussed above, research ethics committees must review the need for special protection of their rights and welfare"(p.58).* The implementation of special protections are listed generally but apply to all vulnerable groups (not specific groups) *SPECIFICALLY “Women in general must not be considered vulnerable, specific circumstances in which women could be vulnerable in research include studies with female refugees and asylum seekers and research with women who live in a cultural context where they are not permitted to consent on their own behalf for participation in research, but require permission from a spouse or male relative.” (p. 58)*
HANDBOOK FOR GOOD CLINICAL RESEARCH PRACTICE (GCP) GUIDANCE FOR IMPLEMENTATION (WHO GCP) *(WHO)*	2002	Int	WHO Good Clinical Practice	"*All individuals, including healthy volunteers, who participate as research subjects should be viewed as intrinsically vulnerable.” (p.22).*	Takes a broad approach to vulnerability in that all individuals, who participate as research subjects are vulnerable but [groups] *"such as children, prisoners, pregnant women, handicapped or mentally disabled persons, or economically or educationally disadvantaged persons are likely to be more vulnerable." (p. 22)*	*There may be factors – social, cultural, economic, psychological, medical – that adversely affect the subjects’ ability to make rational, objective choices to protect their own interests, but may not be apparent to the researcher." (p. 65)*	*GENERALLY* Broad safeguards or protections are suggested but not all are relevant to all vulnerable groups. Describes why special protections are required. *“One special instance of injustice results from the involvement of vulnerable subjects.* *SPECIFICALLY* Justice *- Certain groups, such as racial minorities, the economically disadvantaged, the very sick, and the institutionalized may continually be sought as research subjects, owing to their ready availability in settings where research is conducted. Given their dependent status and their frequently compromised capacity for free consent, they should be protected against the danger of being involved in research solely for administrative convenience, or because they are easy to manipulate as a result of their illness or socioeconomic condition.” (p.66)*	No
DIRECTIVE 2001/20/EC OF THE EUROPEAN PARLIAMENT AND OF THE COUNCIL of 4 April 2001 (EU Clinical Trails Directive) *(European Commission)*	2001	EU	EU Clinical Trials Directive	No	*"Children represent a vulnerable population." (p.2)*	[Children] *"with developmental, physiological and psychological differences from adults." (p.2)*	*GENERALLY "Criteria for the protection of children in clinical trials therefore need to be laid down." (p.3)*	No
Integrated Addendum to ICH E6(R1): Guideline for Good Clinical Practice (2016) (ICH GCP) *(International Conference on Harmonization)*	1996	US, EU, JP, AUS, CA	ICH Harmonized Guideline	No	*"…..members of a group with a hierarchical structure, such as medical, pharmacy, dental, and nursing students, subordinate hospital and laboratory personnel, employees of the pharmaceutical industry, members of the armed forces, and persons kept in detention. Others include patients with incurable diseases, persons in nursing homes, unemployed or impoverished persons, patients in emergency situations, ethnic minority groups, homeless persons, nomads, refugees, minors, and those incapable of giving consent." (p.8)*	*"Individuals whose willingness to volunteer in a clinical trial may be unduly influenced by the expectation, whether justified or not, of benefits associated with participation, or of a retaliatory response from senior members of a hierarchy in case of refusal to participate." (p.8)*	*GENERALLY "An Institutional Review Board should safeguard the rights, safety, and well-being of all trial subjects. Special attention should be paid to trials that may include vulnerable subjects." (p.10)*	No
***National***								
Tri-Council Policy Statement, Ethical Conduct for Research Involving Humans. Qualitative Research (Chapter 10) (2018) (TCPS2) *(Interagency Advisory Panel on Research Ethics)*	2014	Canada	Tri-Council Statement	*"A diminished ability to fully safeguard one’s own interests.. caused by limited decision-making capacity or limited access to social goods, such as rights, opportunities and power. Individuals or groups may experience vulnerability to different degrees and at different times, depending on their circumstances." (p.210)*	*"Individuals or groups whose circumstances may make them vulnerable in the context of research have historically included children, the elderly, students, women, prisoners, those with mental health issues and those with diminished capacity for self-determination. Ethnocultural minorities and those institutionalized." (p.8).*	*"Because their incapacity to make decisions puts them in circumstances that may make them vulnerable." (p.43)*	*GENERALLY "For vulnerable participants, additional measures are needed to protect their interests and to ensure that their wishes (to the extent that these are known) are respected."* (p.7). *"Respect for Persons requires involving individuals in circumstances of vulnerability in decision making where possible" (p.7)* *SPECIFICALLY* Justice *– “Vulnerability is often caused by limited decision-making capacity, or limited access to social goods, such as rights, opportunities and power such as groups who have, at times, been treated unfairly and inequitably in research, or have been excluded from research opportunities. (p.8)*. or "*some groups have been unfairly included in research because they are convenient populations for research (e.g., prisoners, students, people with limited financial resources or those in other circumstances of vulnerability)" (p.50) "A threat to Justice is the imbalance of power that may exist in the relationship between researcher and participant." (p.8)* Capacity to consent* "...development of consent materials that are appropriate to the cognitive and communication abilities of prospective participants." (p.44)*	No
National Statement on Ethical Conduct in Human Research (National Statement) *(National Health and Medical Research Council)*	2007	Australia	Australian National Statement	No	"*Women who are pregnant and the human foetus, children and young people, people in dependent or unequal relationships, people highly dependent on medical care who may be unable to give consent, people with a cognitive impairment, an intellectual disability, or a mental illness, people who may be involved in illegal activities, Aboriginal and Torres Strait Islander peoples, people in other countries." (p.61)*	*"Children’s particular ethical concerns: their capacity to understand what the research entails, and therefore whether their consent to participate is sufficient for their participation; their possible coercion by parents, peers, researchers or others to participate in research; and conflicting values and interests of parents and children." (p.65)*	*GENERALLY "Ethical considerations specific to participants"* groups are listed *(p.61) GENERALLY -* Where “*potential participants [in dependent or unequal relationships] are especially vulnerable” special measures may be required (p.53)* *SPECIFICALLY* Beneficence *- “People with a cognitive impairment, intellectual disability, or mental illness have distinctive vulnerabilities as research participants”* and are* “more-than-usually vulnerable to various forms of discomfort or stress” (p. 58).* Justice - *"...some people are vulnerable to being over researched because of the relative ease of access to them as research populations. Researchers should take account of this vulnerability in deciding whether to seek out members of these populations as research participants." (p.68)*	*SPECIFICALLY* *"Pre-existing relationships between participants and researchers or between participants and others may compromise the voluntary character of participants’ decisions for example……. government authorities and refugees." (p.68)*
Research Governance Framework for Health and Social Care, 2nd edition 2018 (UK Framework) *(Health Research Authority (HRA))*	2005	UK	UK Research Governance Framework	No	No	No	*SPECIFICALLY* Capacity to consent - *"Care is needed when seeking consent from children and from vulnerable adults, such as those with mental health problems or learning difficulties. Information is provided in appropriate written or pictorial form, and that the role and responsibilities of parents, carers or supporters are clearly explained and understood." (p.7)*	No
Title 45 Code of Federal Regulations, Part 46 (Common Rule) *(US Department of Health and Human Services)*	1991	US	Common Rule	No	*"If an [Institutional Review Board] regularly reviews research that involves subjects that are vulnerable such as children, prisoners, individuals with impaired decision-making capacity, or economically or educationally disadvantaged persons." (p.9)*	*"Research that involves category of subjects that is vulnerable to coercion or undue influence."(p.9)*	*GENERALLY "If an Institutional Review Board regularly reviews research that involves a category of subjects that is vulnerable ...... consideration shall be given to the inclusion of one or more individuals who are knowledgeable about and experienced in working with these categories of subjects"(p.9)* *SPECIFICALLY* Justice - *"Selection of subjects is equitable. Institutional review boards should be particularly cognizant of the special problems of research that involves subjects who are vulnerable." (p.11)*	No
The Belmont Report	1979	US	Belmont report	No	*"… involvement of vulnerable subjects. Certain groups, such as racial minorities, the economically disadvantaged, the very sick, and the institutionalized." (p.9)*	"*In some situations, however, application of the [voluntary] principle is not obvious. The involvement of prisoners for example. The principle of respect for persons requires that prisoners not be deprived of the opportunity to volunteer for research. However, under prison conditions they may be subtly coerced or unduly influenced to participate for which they would not otherwise volunteer." (p.4)*	*GENERALLY "The extent of protection afforded should depend upon the risk of harm and the likelihood of benefit. The judgment that any individual lacks autonomy should be periodically re-evaluated and will vary in different situations" (p.4) " A number of variables go into such judgments [of vulnerable populations], including the nature and degree of risk, the condition of the particular population involved, and nature and level of the anticipated benefits." (p.8)* *SPECIFICALLY* Justice *– “the selection of research subjects needs to be scrutinized in order to determine whether some classes (e.g., welfare patients, particular racial and ethnic minorities, or persons confined to institutions) are being systematically selected simply because of their easy availability, their compromised position, or their manipulability, rather than for reasons directly related to the problem being studied." (p.6)* Justice* -“injustice arises from social, racial, sexual and cultural biases institutionalized in society” (p.9) “Certain groups ... may continually be sought as research subjects, owing to their ready availability in settings where research is conducted. Given their dependent status and their frequently compromised capacity for free consent, they should be protected against the danger of being involved in research solely for administrative convenience, or because they are easy to manipulate as a result of their illness or socioeconomic condition." (p.9)* Capacity to consent* - Comprehension in informed consent "Because the subject's ability to understand is a function of intelligence, rationality, maturity and language, it is necessary to adapt the presentation of the information to the subject's capacities."(p.7) "Special provision may need to be made when comprehension is severely limited." (p.7)*	No

### Describing Vulnerability

All fourteen ethical guidelines referred to “vulnerability” or “vulnerable populations” or “vulnerable groups” but only four defined these terms (Table 1)(World Health Organization 2001; Interagency Advisory Panel on Research Ethics 2014; Council for International Organizations of Medical Sciences 2016; World Health Organization 2011). Most commonly, vulnerability was defined in relation to dimished autonomy and/or provided examples of characteristics considered vulnerable. Definitions related to an increased risk of harm were rare and no guidelines defined vulnerability in relation to the principle of justice.

Of the guidelines that defined vulnerability in relation to the principle of respect for persons, one took a broad view of the principle of respect stating that all research participants are viewed as vulnerable because there may be social, cultural, economic, and psychological factors that adversely affect their ability to make informed choices about participating in research (World Health Organization 2001). Two, defined vulnerability in relation to perceptions of coercion or undue influence to participate in contexts of unequal power (Interagency Advisory Panel on Research Ethics 2014). They pointed out that even in the absence of overt coercion, the decision to participate in research might reflect deference to the researcher’s perceived position of power (National Health and Medical Research Council 2007) and that power imbalances or coercion could affect the relationship with the researcher and decision-making procedures (Interagency Advisory Panel on Research Ethics 2014).

Three guidelines defined vulnerability as participants being incapable of protecting their own interests (Interagency Advisory Panel on Research Ethics 2014; Council for International Organizations of Medical Sciences 2016; World Health Organization 2011). Another inferred vulnerability as the ability to consent voluntarily and be fully informed is compromised (International Conference on Harmonization 1996). Only one guideline acknowledged that vulnerability is circumstantial and is experienced differently depending on a person’s situation (Interagency Advisory Panel on Research Ethics 2014). Three guidelines defined vulnerability in terms of structural disadvantages such as insufficient resources or limited access to social goods such as rights, opportunities, and power (World Health Organization 2001; Interagency Advisory Panel on Research Ethics 2014; Council for International Organizations of Medical Sciences 2016).

One guideline employed a broader concept and considered the context of vulnerability (Council for International Organizations of Medical Sciences 2016). Instead of treating groups as homogenous, this guideline articulated the underlying issues that contributed to vulnerability noting that some features of the circumstances in which people live make it less likely that they will be vigilant about or sensitive to, protecting their own interests. This guideline discussed how the interest of participants as individuals can be protected (Council for International Organizations of Medical Sciences 2016).

One guideline characterized vulnerability in relation to an increased susceptibility to harm (i.e. in relation to the principle of beneficence) (World Medical Association 2001). It defined vulnerable individuals as those who have an increased likelihood of experiencing harm. However, it did not provide further details regarding specific characteristics of who are vulnerable due to their increase susceptibility to harm (World Medical Association 2001). One guideline specifically addressed harm or risk by suggesting that specific populations with diminished comprehension may be more at risk of various forms of stress or discomfort (National Health and Medical Research Council 2007).

### Population Groups/Circumstances Considered Vulnerable and Why

Certain groups or populations were identified as vulnerable in eight of the fourteen guidelines (World Medical Association 2001; World Health Organization 2001, 2011; European Parliament 2001; European Commission Directorate-General for Research and Innovation 2020; US Department of Health and Human Services 2018; National Health and Medical Research Council 2007, Interagency Advisory Panel on Research Ethics 2014). Two other guidelines identified specific characteristics or circumstances which make individuals vulnerable (Interagency Advisory Panel on Research Ethics 2014; Council for International Organizations of Medical Sciences 2016).

A range of vulnerable groups were mentioned. Three identified those with incurable diseases, people in nursing homes, unemployed or impoverished people, patients in emergency situations, homeless people, nomads, and refugees as vulnerable (National Health and Medical Research Council 2007; World Health Organization 2011; International Conference on Harmonization 1996). Two guidelines identified economically or educationally disadvantaged persons and ethnocultural minorities as vulnerable (Interagency Advisory Panel on Research Ethics 2014; U.S. Department of Health and Human Services 2018). One guideline described potential participants in dependent or unequal relationships such as children as especially vulnerable due to the developmental differences between adults and children (European Parliament 2001). Two others suggested people who are institutionalized, economically disadvantaged, or intellectually disabled are also at risk of undue influence or coercion (U.S. Department of Health and Human Services 2018; The National Commission for the Protection of Human Subjects of Biomedical and Behavioral Research 1979). One guideline proposed that students, prisoners, employees, and patients due to being in unequal or dependent relationships with researchers may be unduly influenced by the expectation of benefits associated with participation or of retaliation for refusal to participate (International Conference on Harmonization 1996).

One guideline employed a broader concept and considered the context of vulnerability. Importantly, instead of treating groups as homogenous, this guideline articulated the underlying issues that contributed to certain risks for populations noting that some features of the circumstances in which people live make it less likely that they will be vigilant about, or sensitive to, protecting their own interests. This guideline also discussed how the interests of participants can be protected (Council for International Organizations of Medical Sciences 2016).

Only one guideline characterized vulnerable groups and individuals in relation to an increased susceptibility to harm (i.e. in relation to the principle of beneficence). It defined vulnerable individuals as those who have an increased likelihood of being wronged or incurring additional harm but did not provide further details regarding specific groups or individuals who are vulnerable due to their increased risk (World Medical Association 2001).

### Ethical Considerations Relating to Vulnerable Participants

All fourteen guidelines recommend that vulnerable populations be afforded special protections. However, disparity exists between guidelines with regard to which populations or circumstances they specify as requiring more protection and specifically what protections should be in place.

#### Respect for Persons and Informed Consent

Diminished capacity to consent was the most commonly identified feature for which guidance about special protections was provided on four guidelines (National Health and Medical Research Council 2007; World Health Organization 2001; Interagency Advisory Panel on Research Ethics 2014; Council for International Organizations of Medical Sciences 2016; World Health Organization 2011). One highlighted how individuals who are in unequal power relationships with a researcher have a diminished capacity to consent voluntarily (Council for International Organizations of Medical Sciences 2016). Another proposed that potential participants who are in a subordinate position such as students, workers in settings where research is conducted, and members of the armed forces or police, may be unduly influenced by the benefits associated with participation or of a retaliatory response in case of refusal to participate (World Health Organization 2011). The role of literacy and linguistic ability was also highlighted in one guideline (Interagency Advisory Panel on Research Ethics 2014).

#### Justice and the Fair Section of Participants

Three guidelines addressed the ethical principle of justice when research involves vulnerable populations (National Health and Medical Research Council 2007; Interagency Advisory Panel on Research Ethics 2014; Council for International Organizations of Medical Sciences 2016). Two highlighted that certain groups such as those who are incarcerated, the very sick and the institutionalized, are continually being sought and are in readily available settings and convenient populations to be involved in research due to their circumstances or condition in life (Interagency Advisory Panel on Research Ethics 2014; The National Commission for the Protection of Human Subjects of Biomedical and Behavioral Research 1979). One stated that potential participants with limited decision-making capacity do not have an equal opportunity to participate in research (Interagency Advisory Panel on Research Ethics 2014). Additionally, they suggested an important threat to justice is the imbalance of power between participant and researcher as participants will generally not understand the research in the same way and in the same depth as does the researcher (Interagency Advisory Panel on Research Ethics 2014).

Three guidelines provided detailed but diverse guidance on ethical considerations that need to be addressed when research involves vulnerable participants (National Health and Medical Research Council 2007; Interagency Advisory Panel on Research Ethics 2014; Council for International Organizations of Medical Sciences 2016). These include: Indigenous Australians in dependent relationships (National Health and Medical Research Council 2007). Strategies recommended for protecting these participants included local community engagement to ensure respect for cultural and linguistic diversity (National Health and Medical Research Council 2007), building capacity and emphasizing collective welfare when undertaking research involving the First Nations, Inuit, and Métis Peoples (Interagency Advisory Panel on Research Ethics 2014) and supplementing each participant’s consent by ensuring community or family permission and representation (Council for International Organizations of Medical Sciences 2016).

#### Beneficence (Avoidance of Harm) and Assessment of Risk and Benefits

Canada’s Tri-Council Policy Statement of 2010, Ethical Conduct for Research Involving Humans (TCPS2) provides comprehensive guidance on research with First Nations people with regard to concern for welfare. This guideline states the principle of concern for welfare is broad and requires consideration of the sociocultural and economic context of participants circumstances (Interagency Advisory Panel on Research Ethics 2014). For example, an Indigenous community of interest may designate a local organization to provide advice and ethical protection for a project in which Indigenous people participate. This will help determine any special measures which need to be in place to ensure participants safety in the context of a specific research project.

### Ethical considerations relating to participants from refugee and asylum seeker backgrounds

Four of the fourteen guidelines referred to refugees and recognized the need for research bodies to provide special protection for refugee and asylum seekers (National Health and Medical Research Council 2007; Council for International Organizations of Medical Sciences 2016; World Health Organization 2011; European Commission Directorate-General for Research and Innovation 2020). Two guidelines recognized sources of vulnerability for refugees or asylum seekers which were linked to the principle of respect and diminished autonomy (Council for International Organizations of Medical Sciences 2016; European Commission Directorate-General for Research and Innovation 2020). Women from refugee-like backgrounds where they are not permitted to consent on their own behalf for participation in research, were mentioned and specifically linked to the need to provide informed consent. However, there were no recommendations for how to obtain informed consent from this group (Council for International Organizations of Medical Sciences 2016).

Another guideline recognized the need to consider pre-existing relationships between participants and authorities which may unduly influence and compromise the voluntary nature of participants’ decisions to engage in research. For example, special consideration of the relationship between government authorities and refugees is required (National Health and Medical Research Council 2007).

One guideline, which specifically focused on refugees and asylum seekers, provided a comprehensive overview of how protection can be implemented in practice. It included how to approach informed consent (e.g. signing consent forms may jeopardize their anonymity), incidental findings that may be discovered unintentionally (e.g. such as human and sexual trafficking, forced marriage, or female genital mutilation) and protection of personal data and misuse or disclosing data that may endanger a person (e.g. maintain confidentiality to prevent possible stigmatization, social exclusion or racism)(European Commission Directorate-General for Research and Innovation 2020).

## Discussion

Overall ethical considerations in research with refugees and asylum seekers are not currently addressed in national and international guidelines. Most guidelines defined vulnerability in terms of limitations in people’s capacity to provide informed consent. The lack of guidance on other aspects of refugees and asylum seekers needs might affect ethics committees and researcher’s ability to adequately address the ethical complexities inherent in research with these participants.

### Strengths and Limitations of the Systematic Review

A comprehensive and systematic search was conducted using a published strategy developed by Godin et al. (2015) for searching and retrieving grey literature (Godin et al. 2015). It was supplemented by a hand-search of the International Compilation of Human Research Standards (Office for Human Research Protections 2020) and targeted website searches using Google, an efficient tool for locating organizations’ publications on specific topics (Godin et al. 2015). Selection bias was minimized by having pre-set inclusion criteria.

One limitation was that only English language publications were included and it is possible that relevant papers in languages other than English were missed. The review focused on research with refugees and asylum seekers resettling in high income countries. However, most of the world’s displaced people in 2020 were located in low-and middle-income countries in Africa, Asia, and the Middle East (UNHCR 2020). Additional research ethics considerations are likely to be needed in politically unstable contexts, cross country research settings, and ongoing conflict settings where refugees, asylum seekers and displaced people are particularly vulnerable and volatile and these were not captured.

### Limitations of Guidelines

#### Conceptualization of Vulnerability

As has been highlighted in previous research, we found that ethics guidelines often describe vulnerability in ways that reinforce preconceptions about whole groups of people (Bracken-Roche et al. 2017; Schrems 2014; DuBois et al. 2012; Levine et al. 2004). While categorizing people by group when addressing vulnerability is efficient, it can create harm through stereotyping, creation or perpetuation of prejudice, stigmatization, discrimination, or creating an affront resulting from insensitivity (Thapliyal and Baker 2018). Furthermore, the group-based approach is limiting in situations where a person has multiple vulnerabilities and considerations of individual agency, circumstances or the context of the vulnerability are needed (Gordon 2020).

Furthermore, it has been argued that vulnerability is not a substantive ethical concept in itself, serving only as a marker of other research ethics concerns already captured by existing concepts such as risk of harm or voluntary consent (Wrigley 2015). If these concepts are otherwise missed, categorizing people as vulnerable may prove to be a valuable proxy and serve a practical function by indicating individual circumstances requiring special ethical consideration (Bracken-Roche et al. 2017). Categorization may lead researchers to take particular account of potentially vulnerable people thus signposting individual circumstances, in the context of the study, that place them at increased risk of harm.

As can be seen from our findings, few guidelines identified individual and circumstantial reasons for potential vulnerability (Interagency Advisory Panel on Research Ethics 2014; Council for International Organizations of Medical Sciences 2016). The widely recognized contextual approach (Gordon 2020; Cascio and Racine 2018; Block et al. 2013) allows for a more nuanced understanding of the nature of vulnerability which, in turn helps researchers formulate targeted protections (Childress and Thomas 2018). Circumstances may be personal, specific to a group, or structural in origin and may overlap. For instance, the pre-arrival experiences of refugees or asylum seekers who have mental illness or are incarcerated are generally at risk of harm in more than two ways and have particular needs related to their complex circumstances.

An additional concern is that most guidelines focus on individual, rather than broader social factors and structural coercion that may shape research participation in their advice about how to prevent coercion and ensure participation is voluntary. Since the social, cultural, economic and political contexts influence individual’s decision-making capacity (Fisher 2013), advice about how to address structural factors and avoid coercion needs to acknowledge that people have different adaptive capabilities, agency, needs, priorities and capacities which are not static (Mackenzie et al. 2007; Childress and Thomas 2018). Refugees and asylum seekers may misinterpret the risks and benefits of research participation. Refugees and more specifically those seeking asylum may agree to participate because they incorrectly perceive that it may be beneficial for them. Likely benefits might include granting of refugee status, release from detention or immigration assistance for family members (Gillam 2013). A broader conceptualization of coercion also highlights how power differences between the researcher and participants can intersect to increase the pressure to participate and might help researchers be aware of these and act on them (Childress and Thomas 2018).

While vulnerability is rarely defined, most guidelines implicitly suggest that it is fundamentally an inability to provide free and informed consent. The focus on potential participants’ capacity to provide informed consent is a limitation of existing guidelines. This may limit researchers’ awareness of and ability to address other threats, for example the risk of exploitation (Macklin 2003), or to embrace notions of being harmed and being wronged (Hurst 2008). Furthermore, the COVID-19 pandemic added new complexities to conducting research with populations which have layered vulnerabilities such as people with refugee-like backgrounds. As Salam and colleagues (2022) suggest, with restrictions focusing on maintaining physical distancing set in place to curb the spread of the virus, conducting in-person research and obtaining truly informed consent was further complicated for these communities (Salam, Nouvet, and Schwartz 2022).

One guideline (TCPS2) considers the principle of justice in relation to ethnic minorities (Interagency Advisory Panel on Research Ethics 2014). The TCPS2 states that ethno-cultural minorities are an example of a group who have sometimes been treated unfairly and inequitably in research or have been excluded from research participation opportunities. TCPS2 does recognize that people or groups whose situations make them vulnerable or marginalized may need special attention in order to be treated justly in research. McLaughlin and Alfaro-Velcamp (2015) supports this assertion by suggesting that often, the choices about research participation made by one individual, whose interests may or may not be consistent with those of their community, can influence other people’s decision to take part in a research project (McLaughlin and Alfaro-Velcamp 2015). More recently COVID-19 restrictions have impacted on individuals being recruited into research. For instance, local community organizations may have closed and communication about research with community may be limited and difficult to establish (Salam et al. 2022). An individual’s decision to participate may contribute to unfair distribution of risks such as retaliation from the community and few benefits for people in some refugee communities (Dingoyan, Schulz, and Mosko 2012; Gabriel et al. 2017; Council for International Organizations of Medical Sciences 2016).

TCPS2 also provides some consideration of the principle of beneficence with respect to concern for welfare for First Nations people but does not extend this to immigrant or refugee people. This is a gap in the existing guidelines, as the importance of showing benefits of research has been widely acknowledged (Block et al. 2013; Mackenzie et al. 2007; Brear and Gordon 2021). MacKenzie and colleagues (2007) argue that researchers might move beyond harm minimization and recognize the obligation for researchers to prioritize research projects that promote benefits for refugee participants and their communities (Mackenzie, McDowell, and Pittaway 2007).

#### Linking Ethical Considerations to Participants’ Circumstances or Situation

A key finding was the limited guidance available on ethical considerations specifically aimed at refugees and asylum seekers (National Health and Medical Research Council 2007; Interagency Advisory Panel on Research Ethics 2014; Council for International Organizations of Medical Sciences 2016). This is an important gap as it is widely acknowledged that detailed descriptions of the contexts of vulnerabilities and how they relate to specific ethics principles need to be addressed in research protocols and taken into consideration by ethics committees (National Health and Medical Research Council 2007; Pieper and Thomson 2016; Bracken-Roche et al. 2017). The role of guidelines is to introduce the broad principles of responsible and accountable research practice and provide ethical norms for research conduct (National Health and Medical Research Council 2007). However, this review highlights the need for supplementary frameworks that provide practical advice about how to operationalize and apply ethical principles in research with refugees and asylum seekers who may face particular challenges.

### Suggested Refinements to Research Ethics Guidelines

First, we believe that ethical guidelines should refine and expand their concept of vulnerability. A more inclusive and nuanced conception of vulnerability is needed to advance understanding of the meaning of vulnerability in the context of research participation (Rogers et al. 2012). Further, revising and expanding current definitions of vulnerability to acknowledge the circumstances that can expose research individual participants to coercion, harm, or risk has been suggested by Fisher (Fisher 2013). Also, Luna’s (2009) guidance on circumstances related to increased risk of harm uses a conceptual tool to examine a research participant’s environment (Luna 2009). The tool identifies potential risks such as those relating to capacity or social pressure in the consent process and suggests targeted strategies for their remediation. It allows for identifying layers of vulnerability and shows how they are expressed and can interact with the research context.

Conceiving vulnerability in this layered way leads to multiple approaches, each addressing different layers. It suggests that multiple answers should be sought and that these should be in accordance with more subtle evaluations of what constitutes risk in research. This may help researchers consider different kinds of protection regarding the kind of layer involved. For example, the illiteracy layer can be addressed by offering two or three short sessions informed consent and working with illustrations. This suggestion was highlighted in only one guideline which recommended the use of consent materials appropriate for the level of literacy and comprehension of potential participants (Interagency Advisory Panel on Research Ethics 2014).

Second, refugees and asylum seekers were only mentioned as populations needing enhanced protections in four of the fourteen guidelines (National Health and Medical Research Council 2007; Council for International Organizations of Medical Sciences 2016; World Health Organization 2011; European Commission Directorate-General for Research and Innovation 2020). Ethics committees and researchers working with refugees and asylum seekers need guidance on how to best protect these participants considering their complex circumstances and the specific challenges of structural coercion. Lange et al. (2013) suggests that identifying different sources of vulnerability generates distinct obligations on the part of the researcher (Lange et al. 2013). For instance, it is important for researchers not to generate or exacerbate a person’s dependency on others, especially dependency on those who are in a position to withhold support. To do so would increase a person’s risk of exploitation or domination by others and might increase their sense of powerlessness. In another example, outlined by Palmer and colleagues (2011), research that seeks to identify and reduce the risk of harm for people who are prone to depression or at threat of domestic violence is often justified by the potential benefits compared with the risks of individual harm.

However, researchers have an obligation to be aware of and limit potential adverse effects of participation including exacerbating feelings of embarrassment, shame, isolation, and an inability to care for one’s family (Palmer et al. 2011). Luna goes further by suggesting researchers and ethics committees can only identify participants who are at risk of harm by carefully examining the characteristics of the potential participants in conjunction with the nature of the proposed research (Luna 2009). Refugees and asylum seekers may be vulnerable to real or perceived threats when participating in research particularly in the early resettlement period in a host country. For example, as Gillam (2013) suggests immigration detention centres remove autonomy of those seeking asylum who are detained and suggests researchers consider whether it is possible to conduct research in a setting where autonomy is not respected (Gillam 2013). Cascio and Racine (2018) also support an individualized person-oriented research ethics approach that considers needs, preferences, or priorities that might impact a person’s risk of harm when participating in research (Cascio and Racine 2018). In order to facilitate this, for example, The National Statement provides advice by suggesting an advocate or support person when potential participants are considering taking part in research (National Health and Medical Research Council 2007).

Furthermore, there are many assumptions about the potential risk of harm for refugees and asylum seekers participating in research. Gillam (2013) suggests that the role of researcher includes investigating the experiences participants have in the research process and the impacts it has on them (Gillam 2013). To date minimal research has been undertaken exploring the effect on refugees and asylum seekers of participating in research (Dyregrov, Dyregrov, and Raundalen 2000; Gabriel et al. 2017). A small number of studies indicate there are therapeutic benefits for refugees and asylum seekers (McMichael and Gifford 2009; Puvimanasinghe et al. 2019). However, there is scarce published evidence on the harmful effects of participation for these groups. As Gillam (2013) suggests, collecting information from studies involving refugees and asylum seekers provides the opportunity to gather data on the effects of research participation and enhance evidence on participation (Gillam 2013). Further research in this field might be used to develop evidence-based guidelines and practice.

This reinforces the need for a comprehensive supplementary refugee-specific research ethics framework as identified by Seagle and colleagues (Seagle et al. 2020). We agree that a practical framework addressing characteristics or circumstances that may contribute to refugees and asylum seekers’ need for special protections is warranted. The National Health and Medical Research Council’s Ethical Conduct in research with Aboriginal and Torres Strait Islander peoples (2018) is an example of what is being proposed for research with refugees and asylum seekers (National Health and Medical Research Council 2018). These ethics guidelines provide a set of specific principles to ensure research is safe, respectful, responsible, high quality, of benefit to Aboriginal and Torres Strait Islander people and communities. By applying the concepts of spirit and integrity, cultural continuity, equity, reciprocity, respect, and responsibility, these guidelines provide greater recognition of the ethical considerations and additional protections required for this group (National Health and Medical Research Council 2018). 

A supplementary refugee and asylum seeker specific framework might include advice about how to assess and manage the special protections needed relating to their experiences, cultural and linguistic differences, and participants’ potential limited English language proficiency and lack of familiarity with research processes. Advice about how these challenges might increase the risk of research-related harms, affect capacity to give informed consent, and participants’ understanding of the planned research activities is also needed. Such a framework might also address the individual contexts of agency, resilience, and skills of people with refugee and asylum seeker backgrounds and how these can be acknowledged, reaffirmed, and strengthened.

A supplementary ethics framework could emphasize the need for researchers to ensure the research question and methods can be made appropriate for people from refugee-like backgrounds contributing data to research. It should also offer guidance on appropriate forms engagement with refugee communities and individuals who participate in research. Guidelines for research with Indigenous Australian people (National and Medical Research 2003) provide guidance on ethical research conduct with this population and may be equally applicable to or provide a model for research with refugee participants. Examples of culturally and linguistically sensitive recommendations for research with refugee participants could include: 1) negotiating the practicalities of participation such as the steps that will be taken to discuss informed consent within the community; 2) the sharing of information about the research process in community languages beyond research participants to the community as a whole; 3) establishing processes that incorporate the needs and rights of both communities and individuals; 4) establishing a community advisory group and respecting the community’s decisions regarding the way the research is to be conducted from project conception to conclusion; and 5) being guided by communities’ cultural norms and suggested methodologies when developing research proposals, where appropriate. These examples advocate refugee centred ethics practices by empowering individuals and communities so they may harness their own agency and decision-making (Bose 2022).

Further recommendations could endorse active and equal participation for refugee communities and individuals in codesigning the research process, facilitating trust-building, and enabling inclusive and respectful conduct of research (Filler et al. 2021). For instance, participants may prefer same gender researchers, particularly for research involving sensitive topics. The researcher’s nationality may be a matter of concern for others in certain political contexts (Gabriel et al. 2017). Establishing participants history of trauma and background experiences is also recommended so that their participation in research is meaningful and beneficial for trauma-exposed participants (Jefferson et al. 2021). Ascertaining previous research experience, fear of consequences of refusing to participate, and fear of loss of confidentiality (Gabriel et al. 2017) particularly for asylum seekers are key recommendations for inclusion in guidelines.

### Strengths and Limitations of the Systematic Review

A comprehensive and systematic search was conducted using a published strategy developed by Godin et al. (2015) for searching and retrieving grey literature (Godin et al. 2015). It was supplemented by a hand-search of the International Compilation of Human Research Standards (Office for Human Research Protections 2020) and targeted website searches using Google, an efficient tool for locating organizations’ publications on specific topics (Godin et al. 2015). Selection bias was minimized by having pre-set inclusion criteria.

One limitation was that only English language publications were included and it is possible that relevant papers in languages other than English were missed. The review focused on research with refugees and asylum seekers resettling in high income countries. However, most of the world’s displaced people in 2020 were located in low-and middle-income countries in Africa, Asia and the Middle East (UNHCR 2020). Additional research ethics considerations are likely to be needed in politically unstable contexts, cross country research settings, and ongoing conflict settings where refugees, asylum seekers, and displaced people are particularly vulnerable and volatile and these were not captured.

## Conclusion

Current national and international ethics guidelines are limited in their scope and advice relating to the ethical complexities of conducting research in vulnerable populations, including refugees and asylum seekers. To ensure members of ethics committees and researchers consider the potential challenges of conducting research with these groups, guidelines may need to be supplemented with a refugee and asylum seeker specific research ethics framework. Such a framework might include consideration of how circumstances and context can lead to refugees and asylum seekers being vulnerable and advice about how they can best be protected. Consideration of refugee and asylum seeker background and previous research experiences might illuminate how this could impact on future research participation for this group. Advocating for refugee centred ethics practices by empowering individuals and communities so they may harness their own agency and decision-making is also warranted.

## Data Availability

The datasets used and/or analysed during the current study are available from the corresponding author on reasonable request.
